# Incidence of epidural haematoma and neurological injury in cardiovascular patients with epidural analgesia/anaesthesia: systematic review and meta-analysis

**DOI:** 10.1186/1471-2253-6-10

**Published:** 2006-09-12

**Authors:** Wilhelm Ruppen, Sheena Derry, Henry J McQuay, R Andrew Moore

**Affiliations:** 1 Pain Research and Nuffield Department of Anaesthetics, University of Oxford, Oxford Radcliffe NHS Trust, The Churchill Headington, Oxford, OX3 7LJ, UK; 2 University Hospital Basel, Department Anaesthesia, CH-4031 Basel, Switzerland

## Abstract

**Background:**

Epidural anaesthesia is used extensively for cardiothoracic and vascular surgery in some centres, but not in others, with argument over the safety of the technique in patients who are usually extensively anticoagulated before, during, and after surgery. The principle concern is bleeding in the epidural space, leading to transient or persistent neurological problems.

**Methods:**

We performed an extensive systematic review to find published cohorts of use of epidural catheters during vascular, cardiac, and thoracic surgery, using electronic searching, hand searching, and reference lists of retrieved articles.

**Results:**

Twelve studies included 14,105 patients, of whom 5,026 (36%) had vascular surgery, 4,971 (35%) cardiac surgery, and 4,108 (29%) thoracic surgery. There were no cases of epidural haematoma, giving maximum risks following epidural anaesthesia in cardiac, thoracic, and vascular surgery of 1 in 1,700, 1 in 1,400 and 1 in 1,700 respectively. In all these surgery types combined the maximum expected rate would be 1 in 4,700. In all these patients combined there were eight cases of transient neurological injury, a rate of 1 in 1,700 (95% confidence interval 1 in 3,300 to 1 in 850). There were no cases of persistent neurological injury (maximum expected rate 1 in 4,600).

**Conclusion:**

These estimates for cardiothoracic epidural anaesthesia should be the worst case. Limitations are inadequate denominators for different types of surgery in anticoagulated cardiothoracic or vascular patients more at risk of bleeding.

## Background

Epidural anaesthesia and analgesia are widely and successfully used to alleviate perioperative pain. The technique claims to offer many advantages, such as improved cardio-pulmonary function, less intraoperative anaesthetic, improved postoperative gut function, early tracheal extubation, and better mobilisation. There is concern, however, about its use in patients with perioperative anticoagulation, because of the risk of bleeding, which could cause serious adverse events like epidural haematoma and neurological injury.

While potential benefits of epidural anaesthesia and analgesia may be immediate and well reported, information about rare adverse events is more difficult to come by. Because serious harm is uncommon, even large cohorts may report no events. When catheters are used for a short time, as in obstetrics, risks have been calculated [1]: epidural haematoma 1 in 168,000 women, deep epidural infection 1 in 145,000, persistent neurological injury (lasting more than one year) 1 in 240,000, and transient neurological injury (lasting less than one year) 1 in 6700. On the other hand, one person in 35 having a long-term epidural catheter for an average of 70 days for relief of cancer pain can be expected to have a deep epidural infection (unpublished results from a systematic review).

Using epidural catheters in cardiovascular anaesthesia is likely to present risks somewhere between these extremes. The aim of this study was to determine the rates of epidural bleeding and neurological injury for chronic epidural indwelling catheters from available cohorts in cardiovascular and thoracic surgery. This updates a previous review by Ho and colleagues [2] based on 4,600 reported cases of epidural anaesthesia up to 1999 in which no events had occurred. The outcome in this study [2] was the number of cases of epidural anaesthesia in cardiovascular surgery; it so happened that no serious adverse events were reported. We chose purposefully to look for cohorts that positively reported whether events had occurred or not. The difference is no report of an event (a passive approach), and the report of no events (an active approach).

## Methods

**Figure 1:**
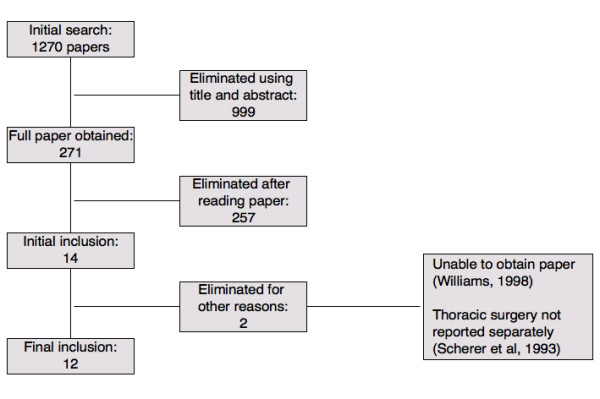
Flow diagram of selection of studies for inclusion for data analysis.

We searched for studies reporting adverse events of cardiovascular anaesthesia in PubMed (from 1966), EMBASE (from 1980), and MEDLINE (from 1966) to February 2005, with no restrictions for language or type of study [1]. Five journals (Anesthesiology, Anesthesia and Analgesia, British Journal of Anaesthesia, Anaesthesia, Acta Anaesthesiologica Scandinavica) were hand-searched from mid-1999 to 2005. Reference lists were checked for additional studies (Figure 1).

Full paper copies were obtained for all studies not eliminated after reading title and abstract. We then selected those reporting on at least 100 cardiovascular or thoracic surgery patients, and with numerical data for serious adverse effects such as haematoma and neurological injuries. This was an arbitrary limit, and previous work has shown that most information on patients comes from larger studies, with small studies contributing a small proportion of patients overall [1]. We took definitions of adverse events as described by the authors of the individual studies. For neurological injuries, we were interested in those that were transient (resolved within one year), and persistent (not resolved within one year).

Information about the type of study, patients, intervention, and numbers experiencing individual adverse outcomes was tabulated. We did not use quality-scoring systems. QUOROM guidelines were followed where applicable. It was the intention, provided there was sufficient clinical homogeneity, to pool results and calculate an overall complication rate using the exact binomial method to determine the 95% confidence interval [3]. For these calculations, a study was included only if it mentioned an adverse event was present or definitely not present; studies not mentioning the adverse event were omitted from that particular calculation. We intended to perform sensitivity analyses if there was sufficient information, for instance for larger versus smaller studies, or more recent versus older studies.

## Results

We identified a large number of papers (1270), with an eventual reference list of 271 papers relating to epidural harm (Figure 1). Fourteen [4-17] related to cardio-vascular-thoracic surgery patients. We could not obtain a copy of one paper [17]. We excluded another [12] reporting on a large series of thoracic and thoracic-abdominal patients because it did not describe results separately. Twelve studies [4-11,13-16] met our search criteria [see Additional file 1]. Five studies [4,5,13-15] (42% of studies but only 14% of patients) were identified by electronic searches, one [6] (8% of studies; 31% of patients) by hand searching, and six [7-11,16] (50% of studies; 55% of patients) by examination of reference lists and reviews.

Three [4,7,10] of the 12 studies concerned vascular surgery and epidural use, and eight [5,8-10,13-16] cardiac surgery, and one [6] included mostly thoracic surgery patients (4,108 thoracic and 245 cardiac). There were 14,105 patients in total, of whom 5,026 patients (36%) had vascular surgery, 4,971 patients (35%) cardiac surgery, and 4,108 patients (29%) thoracic surgery.

All cardiac patients were fully heparinised, and 4,054 of the 4,108 patients (99%) undergoing thoracotomy [6] typically received heparin 5000 U subcutaneously two hours before surgery and then every 12 hours while immobilized. All three studies reporting on vascular surgery [4,7,10] reported extensive anticoagulation of patients.

### Epidural haematoma

There were no cases of epidural haematoma in 4,971 cases of cardiac surgery, 4,108 cases of thoracic surgery, and 5,026 cases of vascular surgery (Table 1).

**Table 1:** Events found in different patient groups

**Author**	**Surgery**	**Year**	**Patients**	**Transient neurological events**	**Persistent neurological events**	**Epidural haematoma**
Baron [4]	Vascular	1987	912	0	0	0
Odoom [7]	Vascular	1983	950	0	0	0
Rao [10]	Vascular	1981	3164	4	0	0
Horlocker [6]	Thoracic	2003	4108	0	0	0
Chakravarthy [16]	Cardiac	2005	2113	4	0	0
Horlocker [6]	Cardiac	2003	245	0	0	0
Pastor [9]	Cardiac	2003	714	0	0	0
Canto [5]	Cardiac	2002	305	no data	0	0
Oxelbark [8]	Cardiac	2001	250	0	0	0
Scott [13]	Cardiac	2001	408	0	0	0
Warters [15]	Cardiac	2000	278	no data	no data	0
Sanchez [11]	Cardiac	1998	558	0	0	0
Turfrey [14]	Cardiac	1997	100	no data	0	0

**Total**			**14105**	**8**	**0**	**0**

### Neurological injury

Ten studies [4,6-12,16] (13,422 patients) reported on transient neurological injury, with eight cases described in two of them [10,16] (Table 1). This was a risk of 8 in 13,422 patients (0.06%, 95% confidence interval 0.03% to 0.12%; a rate of 1 in 1,700, 95% confidence interval 1 in 3,300 to 1 in 850).

None of the eleven studies [4-11,13,14,16] (13,827 patients) reporting on permanent neurological injury found any persistent neurological injury.

## Discussion

Results concerning postoperative morbidity with perioperative epidural analgesia have been contradictory. Some studies [18,19] found an improved outcome for patients with thoracic epidural analgesia, whereas others [20-22] did not find any difference between thoracic epidural analgesia and systemic analgesia [12]. Kehlet & Wilmore [23] included neuraxial blockade as one of the key elements for accelerated recovery from surgery, though mainly in the context of visceral rather than cardiac or thoracic surgery.

In cardiovascular surgery there is some evidence that epidural anaesthesia and analgesia improves haemodynamic stability [24-27], coronary perfusion [28-30], ventricular function [31-33], pulmonary function [33-35], intense analgesia [27,33-38], early tracheal extubation [28,33-35,40,41], and metabolic profile [24,26], and decreases ischemia [28] and the incidence of arrhythmias [14]. On the other hand, Hemmerling [39] found no difference in fast track extubation or in haemodynamic stability between the use of epidural analgesia or systemic opioid analgesia, and Samama [42] suggested in a review that epidurals should not be used in vascular surgery patients.

A meta-analysis [43] of the effects of perioperative central neuraxial analgesia on outcomes after coronary artery bypass surgery (15 studies, 1178 patients) reported no reduction in mortality or myocardial infarction after thoracic epidural analgesia compared with general anaesthesia, though a small reduction might be difficult to see with this limited number of patients. However, there were significant reductions in the risk of dysrrhythmias, pulmonary complications, time to tracheal extubation, and pain scores at rest and with activity. A recent review [44] of intrathecal and epidural anaesthesia and analgesia for cardiac surgery, suggests that while they provide enhanced postoperative analgesia, a "clinically important effect on morbidity and mortality" has not yet been demonstrated.

Any benefits from epidural analgesia have to be balanced by the potential for harm, which might be serious. In 2004 Rosen et al [45] described a favourable outcome in the first case of an epidural haematoma in an adolescent patient after cardiac surgery. Martinez-Palli et al [46] also reported an epidural haematoma in a patient receiving epidural analgesia for vascular surgery. As case reports, these studies had no denominator, so no estimate of adverse event rate could be calculated.

More than 800,000 patients have coronary artery bypass surgery a year [43], and even low adverse event rates could give rise to a significant number of patients harmed. The best estimates we could find for cardiovascular patients with epidural analgesia/anaesthesia, were no cases of epidural haematoma in 14,105 patients, eight cases of transient neurological injury in 13,422 patients, and no cases of persistent neurological injury in 13,827 patients.

According to the mathematical model of the "rule of 3" by Hanley and Lippman-Hand [47], when there are no events the 95% confidence interval that the event will not occur 1 in the total divided by three (n/3). Where no events have occurred, as here with epidural haematoma, the estimate of maximum rate is entirely dependant on the size of the denominator; with no events in 1000 patients the maximum risk is 1 in 333, becoming 1 in 3,333 in 10,000 patients.

Using the rule of 3 the maximum risk for these epidural haematoma therefore becomes 14,105 divided by 3, or 0.02%, or 1 in 4,700 patients. For transient neurological injury we found eight cases in 13,422, or 0.06%, or 1 in 1,700 patients. Again using the Hanley and Lippman-Hand formula for persistent neurological injury where there were no events in 13,827 patients, we might expect a maximum rate of 0.02% or 1 in 4,600 patients.

Because of the apparent simplicity of the rule of 3, we also used the exact binomial calculation [3] to estimate an upper confidence interval for the proportion when there were no events. The inverse of this proportion becomes a frequency. The comparison between the two methods is shown in Table 2. The exact method produced a slightly higher risk estimate, but not with any clinical significance given that there had been no events. The rule of 3 has the advantage of being amenable to mental arithmetic, or at worst a simple calculator.

**Table 2:** Comparison of rule of 3 and exact binomial estimates of risk

	**Number of**		**Lower 95% of risk**	
	
**Outcome**	**Events**	**Patients**	**rule of 3**	**Exact CI**
Epidural haematoma				
Cardiac	0	4971	1 in 1,700	1 in 1,350
Thoracic	0	4108	1 in 1,400	1 in 1,100
Vacular	0	5026	1 in 1,700	1 in 1,350
Combined	0	14105	1 in 4,700	1 in 3,800

Permanent neurological injury				
Combined	0	13827	1 in 4,600	1 in 3,750

We have assumed that the underlying risk for epidural haematoma or persistent neurological injury is about the same in cardiac surgery, thoracic surgery, and vascular surgery. Others may disagree. In that event we use the smaller denominators for each group separately, generating maximum risks of 1 in 1,700, 1 in 1,400 and 1 in 1,700 for epidural anaesthesia in cardiac, thoracic, and vascular surgery respectively using the rule of 3 (Table 2). The fact that these risks look similar is an accident of having similar numbers of patients (4,900, 4,100, and 5,000) for the denominator.

Because the previous attempt to estimate the risk of haematoma with epidural anaesthesia in cardiac surgery used a denominator of 4,600 patients, the result for cardiac surgery is similar to our estimate. The difference in approach is important. Ho and colleagues [2] sought the number of cases of epidural anaesthesia in cardiac surgery, and assumed that the lack of any report of epidural haematoma was the same as no event. This depends on the complete reporting of events, yet there is abundant literature to show that serious adverse events are grossly under-reported in situations as disparate as paediatric intensive care [48] and acupuncture [49]. Under-reporting may be influenced by a variety of factors, including litigation.

Our approach was to look only for larger studies with a positive report of either events or lack of them (showing that they had been looked for), and where there was both a nominator (the number of events) and a denominator (the number of cases). Case reports or case series without a denominator tell us only that serious adverse events can happen; they cannot address the rate at which they happen.

A possible limitation is that we limited our search to studies of 100 patients. This was for practical reasons. We reasoned that few cohort studies with both nominator and denominator would be likely to have been done, and previous experience in obstetric anaesthesia [1] showed that 85% of cases were found in larger studies (in that case 10,000 women or more). We also know that observational studies are difficult to find electronically [1,50], and personal experience is that requests for additional information are rarely helpful. Although we could probably have increased denominators by considerable additional searching or including any smaller studies that may exist, it is unlikely that any substantial difference would have resulted.

## Conclusion

The estimates presented here for cardiothoracic epidural anaesthesia should be the worst case. It is limited by inadequate denominators for different types of surgery in anticoagulated cardiothoracic or vascular patients more at risk of bleeding. The maximum risks for epidural haematoma were estimated as 1 in 1,700, 1 in 1,400 and 1 in 1,700 for epidural anaesthesia in cardiac, thoracic, and vascular surgery respectively, or 1 in 4,700 if we are content that the risk in these three types of surgery are sufficiently similar to combine them.

## Competing interests

The author(s) declare that they have no competing interests.

## Authors' contributions

RAM, HJM, and WR were involved with the original concept and planning the study. WR and SD did data extraction and analysis. WR and RAM prepared the initial manuscript, and all authors read and approved the final manuscript.

## Pre-publication history

The pre-publication history for this paper can be accessed here:



## Supplementary Material

Additional file 1 Details of included papers. Details of the studies, including the number of patients, the indication for surgery, study design, details of methods and main results. Click here for file
