# Supplementary material for: Incidence of epidural haematoma and neurological injury in cardiovascular patients with epidural analgesia/anaesthesia: systematic review and meta-analysis
Source: BMC Anesthesiol. 2006 Sep 12;6:10. doi: 10.1186/1471-2253-6-10 (PMC1586186; doi:10.1186/1471-2253-6-10)
Supplement: Additional file 1 — Details of included papers. Details of the studies, including the number of patients, the indication for surgery, study design, details of methods and main results. [file 1471-2253-6-10-S1.pdf]

Addutional file 1: Details of included papers

Author	Patient number	Patient group / Surgery indication	Study description	Methods	Results
Baron 1987	912	Vascular patients: claudication or limb threatening ischemia	Retrospective	1977 to 1982<br>Patients received continuous epidural catheter. During a portion of the surgical procedure all the patients were transiently totally heparinized.	Epidural haematoma: 0<br>Permanent neurological injury: 0
Odoom 1983	950	Vascular surgery: Reconstructive vascular surgery	Retrospective	1976 to 1980<br>All patients receiving pre-operative oral anticoagulation therapy. Upon arrival in the induction room, thrombotest, partial thromboplastin time and kaolin cephalin clotting time were determined. After induction of anaesthesia an epidural catheter was introduced with a test-dose.<br>Before arterial cross clamping heparin was injected intraarterially, then 250-300 ug heparin/min for 0.5 to 3 hours. Duration of the catheter 48 hours. Three months later full neurological examination.	Epidural haematoma: 0<br>Permanent neurological injury: 0
Rao 1981	3164	Vascular surgery: lower extremity peripheral vascular surgery	Prospective	1973-1978<br>Continuous epidural anaesthesia. Upon arrival in the operating room, introduction of an epidural catheter. If blood was aspirated the patient was rescheduled for surgery under general anaesthesia the following day.<br>Intraoperatively, 50 to 60 minutes after performing the regional block, incremental doses of 500 U of heparin every 3 minutes. Repetition of the heparin dose after 6 hours.<br>24 hours after insertion, catheter was removed one hour before the maintenance dose of heparin.	Epidural haematoma: 0<br>Permanent neurological injury: 0<br>Transient neurological injury: 4<br><br>Four patients experienced paraesthesias in the thigh and leg 3 to 4 days following removal of the catheter. Resolved completely within 3 weeks.
Horlocker 2003	4298	Mixed: cardiac surgery and thoracic surgery:<br><br>4108 (94.4%) procedures primarily thoracic, 245 (5.6%) primarily cardiac.	Prospecitve data collection, but retrospective data analysis	1991 to 1997<br>Catheters placed immediately after induction and tracheal intubation or on completion of the surgical procedure, before emergence.<br>The mean duration of epidural analgesia was 2.4±0.8 days (range, 0–10.7 days). There were no neurologic complications, including spinal haematoma, epidural abscess or catheter site infections, radicular symptoms, or persistent paraesthesias (95% confidence interval, 0-0.08%). Patients undergoing thoracotomy (4054, or 94.3%) typically received heparin 5000 U subcutaneously 2 hours before surgery and then every 12 hours while immobilized.	Epidural haematoma: 0<br>Permanent neurological injury: 0<br><br>Six patients developed new neurological symptoms or postoperative worsening of a previous neurological condition. In all six patients, formal neurologic consultation concluded that epidural catheterization was not the primary cause of the neurologic deficits

Chakravarthy 2005	2113	Cardiac surgery	Prospective	1991 to 2003<br>Puncture of the epidural the day before.<br>Heparin infusions discontinued 6 hours before performing epidural catheterization.	Epidural haematoma: 0<br>Permanent neurological injury: 0<br>Transient neurological injury: 4
Pastor 2003	714	Cardiac surgery: coronary artery bypass surgery with cardiopulmonary bypass	Prospective	1995 to 2002<br>Inclusion criteria: elective surgery, coagulation tests normal, adequate preoperative suspension (7 days) of antiplatelet drugs (aspirin or clopidogrel).<br>Puncture done with patient awake, 60 minutes before using heparin. Median approach only. Patients with oral anticoagulants: catheter systematically removed before the anticoagulation was effective. Normally 4 days of treatment with the epidural.	Epidural haematoma: 0<br>Permanent neurological injury: 0<br><br>Eleven cases with blood in the needle or in the catheter: If PaO_2 more than 15% lower than systematically, surgery performed (this was so in all 11
Canto 2002	305	Cardiac surgery: heart valve surgery	Prospective	1995 to 2000<br>Patients who had replacement or repair of cardiac valves under combined general and epidural anaesthesia.<br>Inclusion criteria: antiplatelet drugs had to be suspended for at least 7 days preoperatively, normal coagulation tests, elective surgery. Insertion of the epidural catheter immediately before induction for general anaesthesia. Heparin intraoperatively to maintain an activated clotting time >450 second as well as mild hypothermia (30ºC).<br>No blood through the needle during puncture.	Epidural haematoma: 0<br>Permanent neurological injury: 0
Oxelbark 2001	250	Cardiac surgery: coronary artery bypass surgery, redos, aortic surgery	Unclear, probably retrospective	1999 to 2000<br>250 unselected patients. 12 to 24 hours preoperatively, a thoracic epidural catheter was inserted in the thoracic 2/3 or 3/4 intervertebral space and kept in place for 4 to 5 days. Oral anticoagulation stopped 10 days preoperatively. Normothermic bypassing. All patients extubated 10 minutes after skin closure.	Epidural haematoma: 0<br>Permanent neurological injury: 0
Scott 2001	408	Cardiac surgery: coronary artery bypass	Randomised controlled trial	Epidural catheter insertion immediately before surgery.<br>Normal coagulation screening. In case of bloody tap, surgery was postponed for 24 hours. Heparin given to achieve activated clotting time >450 sec. Cardioplegia with 28ºC.<br>Orally Ibuprofen every 8 hours.<br>Epidural catheter for 96 hours.	Epidural haematoma: 0<br>Permanent neurological injury: 0
Warters 2000	278	Cardiac surgery: Coronary artery bypass	Retrospective	No time duration of study indicated<br>Epidural catheters were inserted no less than 1 hour before anticoagulation. In case of bloody tap, surgery was delayed for 24 hours. Catheters removed only if the international normalized ratio was greater than 1.5.	Epidural haematoma: 0<br>Permanent neurological injury: 0

Sanchez 1998	558	Cardiac surgery: coronary artery bypass surgery with cardiopulmonary bypass	Prospective	1993 to 1995.<br>A Tuohy 18G epidural catheter was inserted the day before surgery; not more than 3 unsuccessful puncture attempts. The presence of a small amount of blood during needle or catheter placement was not recorded.<br>No patient was on oral anticoagulation, 403 patients were on antiplatelet therapy which was stopped one week preoperatively. Modest bypass hypothermia of 32ºC. Catheter was left for up to 5 days.	Epidural haematoma: 0<br>Permanent neurological injury: 0
Turfrey 1997	100	Cardiac surgery: routine coronary artery bypass graft surgery	Retrospective	9 month period<br>In total there were 218 patients: 118 without epidural, 100 patients with epidural. Epidural catheter was inserted immediatly before induction of anaesthesia. Patients with abnormal coagulation did not receive an epidural. Aspirin-patients were not excluded.	Epidural haematoma: 0<br>Permanent neurological injury: 0
